# The identification of retro-DNAs in primate genomes as DNA transposons mobilizing via retrotransposition

**DOI:** 10.12688/f1000research.130043.3

**Published:** 2024-05-29

**Authors:** Wangxiangfu Tang, Ping Liang

**Affiliations:** 1 Department of Biological Sciences, Brock University, St. Catharines, Ontario, L2S 3A1, Canada; 2 Centre of Biotechnology, Brock University, St. Catharines, Ontario, L2S 3A1, Canada

**Keywords:** Primates, DNA transposons, Retrotransposons, Retro-DNA, Target-primed reverse transcription

## Abstract

**Background:**

Mobile elements (MEs) constitute a major portion of the genome in primates and other higher eukaryotes, and they play important role in genome evolution and gene function. MEs can be divided into two fundamentally different classes: DNA transposons which transpose in the genome in a “cut-and-paste” style, and retrotransposons which propagate in a “copy-and-paste” fashion via a process involving transcription and reverse-transcription. In primate genomes, DNA transposons are mostly dead, while many retrotransposons are still highly active. We report here the identification of a unique group of MEs, which we call “retro-DNAs”, for their combined characteristics of these two fundamentally different ME classes.

**Methods:**

A comparative computational genomic approach was used to analyze the reference genome sequences of 10 primate species consisting of five apes, four monkeys, and marmoset.

**Results:**

From our analysis, we identified a total of 1,750 retro-DNAs, representing 748 unique insertion events in the genomes of ten primate species including human. These retro-DNAs contain sequences of DNA transposons but lack the terminal inverted repeats (TIRs), the hallmark of DNA transposons. Instead, they show characteristics of retrotransposons, such as polyA tails, longer target-site duplications (TSDs), and the “TT/AAAA” insertion site motif, suggesting the use of the L1-based target- primed reverse transcription (TPRT) mechanism. At least 40% of these retro-DNAs locate into genic regions, presenting potentials for impacting gene function. More interestingly, some retro-DNAs, as well as their parent sites, show certain levels of expression, suggesting that they have the potential to create more retro-DNA copies in the present primate genomes.

**Conclusions:**

Although small in number, the identification of these retro-DNAs reveals a new mean for propagating DNA transposons in primate genomes without active canonical DNA transposon activity. Our data also suggest that the TPRT machinery may transpose a wider variety of DNA sequences in the genomes.

## Introduction

Mobile elements (MEs), also known as transposable elements, collectively constitute significant portions of the genomes for most higher organisms, being around 50% for primates. ^1^^–^^4^ Despite being initially considered “junk” DNA, research from the last few decades has demonstrated that MEs make significant contributions to genome evolution and impact gene function via a variety of mechanisms. These mechanisms include, but are not limited to, generation of insertional mutations and genomic instability, creation of new genes and splicing isoforms, exon shuffling, and alteration of gene expression and epigenetic regulation. ^5^^–^^17^

Based on the type of the transposition intermediate, MEs can be divided into two major classes: Class I, called “retrotransposons”, that utilize an RNA-intermediate to transpose in a “copy-and-paste” fashion, and Class II termed “DNA transposons”, that work directly at the DNA level to transpose in a “cut-and-paste” style. Furthermore, despite both having target site duplications (TSDs), the two ME classes differ in sequence characteristics, including consensus sequences unique to each class/subclass, distinct TSD length profile, and presence or absence of terminal inverted repeats (TIRs) or polyA tail, and others. ^17^^–^^19^

Retrotransposons represent the majority of MEs in primate genomes, owing to their “copy-and-paste” style transposition, which results in direct copy number increase over time, conjugated with their continuing activity over the course of evolution up to the current time. In this process, a retrotransposon is first transcribed into RNA, which is then reverse-transcribed into DNA as a new copy inserting into a new location in the genome. ^20^ Retrotransposons can be divided into two major subtypes: the long terminal repeats (LTR) and non-LTR retrotransposons, with the former carrying two LTRs flanking the internal viral sequences, while the latter lack LTRs but mostly carry a polyA tail. ^1^ LTRs represent domesticated retroviruses from those infecting the germline cells of the ancestors and becoming integrated into the host genome, and for this reason, they are also called endogenous retrovirus (ERVs). ^21^^,^^22^ In primate genomes, LTRs exist either as full-length LTRs and can be as long as 10kb, or solo-LTRs around 1kb in length as a product of post-insertion homology-based recombination between the two LTRs, which removes the long internal viral sequences. With several hundred thousand copies, LTRs contribute to ~9% of the genomes with a low level of ongoing activity. ^23^^–^^26^

The non-LTR retrotransposons, on the other hand, as the most successful MEs in primate genomes, contribute to more than 35% of the genomes and more than 80% of all MEs in these genomes with several millions of copies. ^3^^,^^4^ From their sequence features, the currently known non-LTR MEs in primate genomes belong to four subclasses, including short-interspersed nuclear elements (SINEs), long-interspersed nuclear elements (LINEs), SINE-R/VNTR/Alu (SVAs), and processed pseudogenes ( *i.e.* retro-copies of mRNAs, also called retro-genes). ^4^^,^^27^^–^^31^ Despite having many differences with regard to their length, consensus sequences, and coding capacity, all subclasses of non-LTR retrotransposons share the common properties of having a 3’-polyA tail and the use of target-prime reverse transcription (TPRT) mechanism for retrotransposition. ^31^^,^^32^ Among them, LINE-1s (L1s) as the only subfamily of autonomous non-LTR retrotransposons in the primate genomes provide the TPRT machinery for all other non-autonomous non-LTR retrotransposons. For this reason, all non-LTR retrotransposons share the same “TT/AAAA” sequence motif at their insertion sites. ^9^^,^^32^^–^^36^

In contrast, DNA transposons, initially known as “jumping genes”, move in genomes using a transposase encoded by autonomous copies. ^1^ Ten out of the twelve DNA transposon superfamilies are known to excise themselves out from their original locations as double-stranded DNA and move to new sites in the genome, which leads to no direct change in their copy numbers. ^17^^,^^19^ Two of the superfamilies, *Helitrons* and *Mavericks*, transpose through non-canonical mechanisms by utilizing a single-stranded DNA as intermediate, which leads to a “copy-and-paste” style. ^17^^,^^37^^,^^38^ The ten “cut-and-paste” DNA transposon superfamilies, as well as *Mavericks*, have TIRs and TSDs, while *Helitrons* is the only superfamily with neither TIRs nor TSDs, owing to its rolling-circle mechanism. ^17^^,^^37^ In addition to these aforementioned DNA transposons, there is another group of DNA transposons named miniature inverted-repeat transposable element (MITEs) characterized by the presence of both TSDs and TIRs yet lacking the coding capacity for the transposase. ^39^ By using DNA transposases encoded by other autonomous DNA transposons, these non-autonomous, short (50-600bp) MITEs can transpose in the host genome. ^17^^,^^40^

DNA transposons have been considered inactive in the current primate genomes and have received very little research attention. Lander *et al.* (2001) in their initial human genome analysis concluded that there was no evidence for DNA transposon activity during the past 50 My, ^2^ while a later study suggested that DNA transposons had been highly active during the early part of primate evolution till ~37 Mya. ^19^ There has been no report for lineage-specific or species-specific DNA transposons in primate genomes. However, in our recent comparative analysis of species-specific MEs in eight primates from the *Hominidae* and the *Cercopithecidae* families, there were also a total of 2,405 DNA transposons identified to be species-specific in addition to the 228,450 species-specific retrotransposons. ^36^ As part of efforts to understand the mechanism(s) underlying these species-specific DNA transposons, we performed further comparative analysis across ten primate genomes and identified a novel group of MEs with the sequences originated from DNA transposons, but showing some hallmarks of L1-based retrotransposons, which we called “retro-DNAs”.

## Results

### Overall profiles of DNA transposons and lineage-specific retro-DNAs in the ten primate genomes

To identify all retro-DNA events in the primate genomes, we first identified the diallelic DNA transposons (da-DNAs) that are defined as DNA transposons with both the insertion allele and pre-integration allele identifiable in one or more of these genomes. These DNA transposons are likely to be the results of relatively recent transposition events shown as having a low level of sequence divergence from their parent copies, which permits accurate identification of TSDs and TIRs. The starting lists of DNA transposons were based on the RepeatMasker annotation subjected to a consolidation process to ensure the accuracy in identifying DNA transposons with both insertion and pre-integration alleles as well as their TSDs. ^3^^,^^36^ One main type of targets for integration in this case are the ME entries split by insertion of other MEs and non-ME sequences. As shown in Table 1, the number of DNA transposons in the primate genomes dropped ~18% on average after integration, leading to less variation in their numbers across genomes ranging from 324,288 in marmoset to 421,580 in chimpanzee, and averaging at 376,720 copies per genome verse 459,521 per genome before integration. These DNA transposons contributed to a total of ~98 Mbp or ~3.6% of these primate genomes on average ( Table 1). Various factors could have contributed to the different DNA transposon numbers in these genomes, including, but not limited to, the differences in the versions of RepeatMasker and the ME reference sequences used for ME annotation, the quality of genome assemblies, and probably most importantly the different evolution history of the individual genomes.

**Table 1.** Summary of DNA transposons in the 10 primate genomes.

Genomes	Raw counts	integrated counts	% count reduction	total size (bp)	% genome	full-length count	% full-length	diallelic DNA counts
**hg38 (human)**	483,994	399,590	17	102,664,356	3.5	119,368	29.9	25,933
**panTro5 (chimp)**	510,250	421,580	17	107,832,154	3.8	119,265	28.3	28,273
**gorGor4 (gorilla)**	503,480	418,454	17	106,573,049	3.8	117,263	28.0	27,386
**ponAbe2 (orangutan)**	429,467	347,471	19	93,420,030	3.4	113,425	32.6	23,923
**nomLeu3 (gibbon)**	438,800	363,738	17	93,531,426	3.6	108,334	29.8	24,206
**macFas5 (crab-eating macaque)**	443,909	359,802	19	94,910,440	3.5	109,444	30.4	26,218
**rheMac8 (rhesus)**	486,991	401,546	18	102,546,356	3.7	111,558	27.8	28,149
**papAnu2 (baboon)**	459,662	369,684	20	97,943,467	3.7	109,523	29.6	25,844
**chlSab2 (green monkey)**	445,724	361,048	19	95,097,218	3.5	108,139	30.0	26,252
**calJac3 (marmoset)**	392,937	324,288	17	83,220,943	3.2	91,946	28.4	34,901
**Average**	459,521	376,720	18	97,773,944	3.6	110,827	29.5	27,109

^*^ Full-length is defined as >=90% of consensus

Using a multi-way comparative genomics approach modified from our previous analysis of human-specific MEs, ^36^ we identified a total of 271,085 da-DNAs in the 10 primate genomes ( Table 1). Specifically, for each da-DNA, we require the presence of a pre-integration allele in at least one of the other nine genomes. As shown in Table 1, the number of da-DNAs varied from 23,923 in the orangutan genome to 34,901 in the marmoset, averaging at 27,109 for the 10 genomes. The largest number of da-DNAs in the marmoset was expected for its largest evolutionary distance from the remaining primate species. Notable differences were also seen between genomes with mutually closest evolutionary relationship among the 10 genomes, making these numbers directly comparable for the paired genomes. For example, between the human and chimpanzee genomes, the latter had >10% more da-DNAs than the former (28,273 *versus* 25,933), while between the two macaques, the rhesus genome had ~10% more than the crab-eating macaque genome (28,149 *versus* 26,218) ( Table 1). In comparison, the species-specific non-LTR retrotransposons in the crab-eating macaque genome were less than 1/8 of that for the rhesus genome (3,039 versus 25,085), ^3^ indicating at least that the lower number of da-DNAs in rhesus genome was not due to genome sequence quality differences.

**Figure 1.**
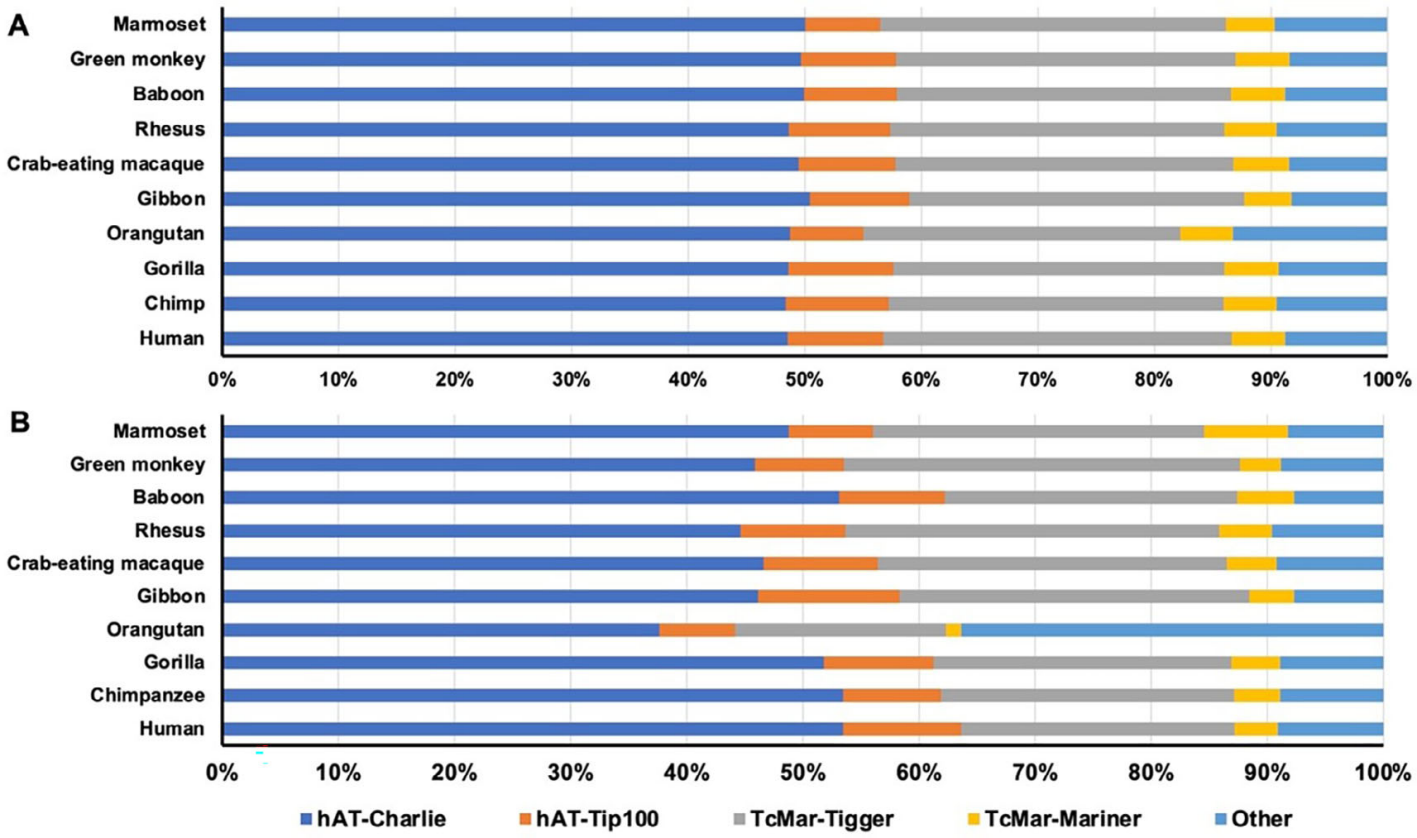
The composition of diallelic DNA transposons and retro-DNAs by family in the ten primate genomes. Horizontal stack bar charts showing the family composition of diallelic DNA transposons (A) and retro-DNAs (B) in each of the 10 primate genomes. The color scheme is the same for both panels.

By composition in DNA transposon type, the majority of the da-DNAs belonged to the *hAT* and *TcMar* superfamilies with the *hAT* subfamilies ( *hAT-Charlie* and *hAT-Tip100*) contributing to ~57% of da-DNAs and the *hAT-Charlie* subfamily alone contributing to ~50% of all da-DNAs in all genomes (Table S1, Figure 1A). The two *TcMar* families, *TcMar-Tigger* and *TcMar-Mariner*, contributed ~33% of da-DNAs, while the remaining families contributed to ~10% of da-DNAs. This composition pattern seems to be quite similar among all genomes, with the orangutan genome having a slightly lower portion from the *hAT-Tip100* and *TcMar-Tigger* families but slightly more from the other families ( Figure 1A, Table S1). In comparison, the family composition for retro-DNAs is shown to be more variable across the genomes, particular in the orangutan genome having lower portion for all 4 named subfamilies but much more from the other (unclassified) class ( Figure 1B).

### Retro-DNAs in the primate genomes possess non-LTR retrotransposon sequence characteristics

**Figure 2.**
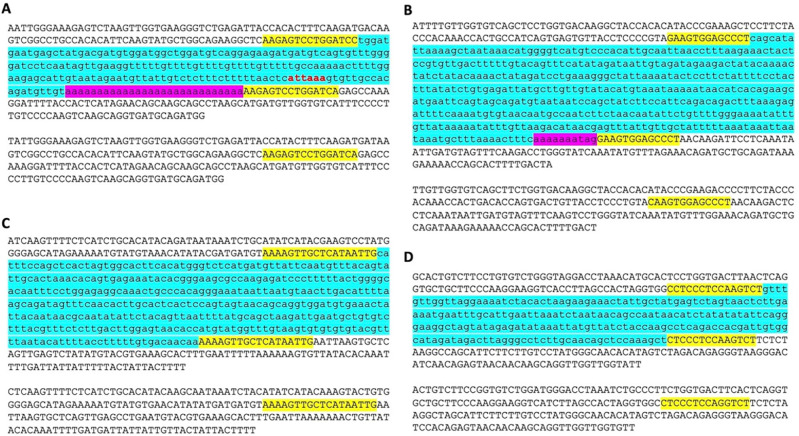
Examples of retro-DNAs in different primate genomes. A. A retro-DNA from the human genome (hg38_chr4:146335052-146335253) with the pre-integration allele from the chimpanzee genome (panTro5_chr4:38758218-38758438). B. A retro-DNA from the green monkey genome (chlSab2_chr8:30005081-30005527) with the pre-integration allele from the gibbon genome (nomLeu3_chr8:37535028-37535236); C. A retro-DNA located from the green monkey genome (chlSab2_chrX:73456937-73457324) with the pre-integration allele from the orangutan genome (ponAbe2_chrX:82896142-82896360). D. A retro-DNA located from the human genome (hg38_chr4:38758216-38758442) with the pre-integration allele from green monkey genome (chlSab2_chr27:11529606-11529817). In each panel, the sequence at the top is the insertion allele containing the retro-DNA and the sequence at the bottom is the pre-integration allele without the retro-DNA. The yellow highlights indicate TSDs, and the blue highlights indicate the DNA transposon sequences, while the purple highlights indicate possible polyA tail sequences.

While analyzing the da-DNAs in detail for understanding the possible mechanisms involved, we came across an unusual case of a 201-bp *Tigger7* DNA transposon from the *TcMar-Tigger* family located at *chr4:146335052-146335253* of the human genome (GRCh38). It appears to be a human-specific ME for its absence in the orthologous region in the chimp genome ( Figure 2A), and more interestingly, this DNA transposon insertion has a 14 bp TSD “AAGAGTCCTGGATC” that is much longer than TSDs for DNA transposons, and it has no identifiable TIR typical of a DNA transposon ( Figure 2A). Furthermore, it has a 27 bp polyA tail at its 3’-end and a predicted polyadenylation signal “ATTAAA” before the polyA tail. All these features point to a non-LTR retrotransposon rather than a canonical *Tigger7* DNA transposon, which is expected to have TIRs and 2 bp (TA) TSDs. We therefore named it as a “retro-DNA” for being a retrotransposon-like element derived from a DNA transposon sequence.

Following the identification of this retro-DNA, we searched the human genome and other primate genomes and identified more similar cases, as exampled in Figure 2B-D. For instance, a 446 bp *Charlie1a* fragment from the *hAT-Charlie* family was identified as a retro-DNA in the genome of three primates (green monkey, rhesus, and crab-eating macaque), which has TSDs in 13 bp long but no TIRs ( Figure 2B).

**Figure 3.**
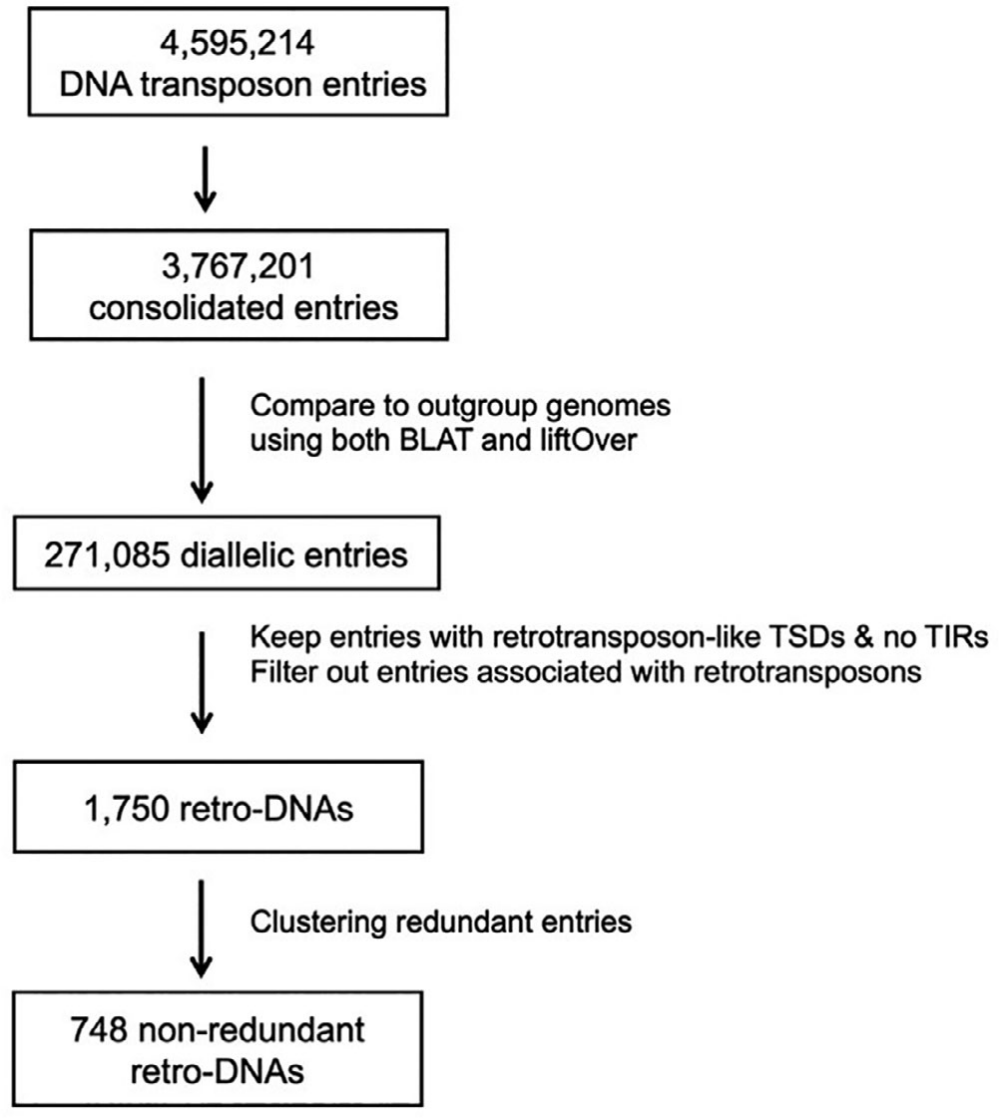
A flow chart for identification of retro-DNAs.

By requiring the presence of longer TSDs (≥8 bp) and the absence of TIRs, we identified a total of 1,750 retro-DNA entries among all da-DNAs using a workflow shown in Figure 3. By classification, these retro-DNAs consist of 847, 478, 156, 74, and 195 entries from the *hAT-Charlie*, *TcMar-Tigger*, *hAT-Tip100*, *TcMar-Mariner*, and other families, respectively ( Table 2). The composition pattern ( Figure 1B) in general was similar to that of all da-DNAs ( Figure 1A), indicating there is no strong bias for retro-DNA towards any particular subfamily among da-DNAs. However, at the genome level, the ratios of retro-DNAs in the orangutan genome from the *hAT-Tip100* and *TcMar-Tigger* families were much lower, while that from the “other” families was much higher compared to other genomes (25% versus 10%) ( Figure 1B). As seen in Table 2, the 1,750 retro-DNAs encompassed all 10 genomes and could be clustered into 748 unique retro-DNA insertion events based on their orthologous relationships. It is worth noting that our list of retro-DNAs may suffer a certain level of false negatives and false positives due to the uses of a set of criteria that might not be optimal and due to the challenges associated with the analysis of MEs and the deficiencies of the reference genome resources, especially for the non-human primates as discussed in our recent study. ^3^

**Table 2.** The distribution of retro-DNAs by subfamilies in the 10 primate genomes.

DNA transposon family	Human	Chimpanzee	Gorilla	Orangutan	Gibbon	Crab-eating macaque	Rhesus	Baboon	Green monkey	Marmoset	Total	Total (nr)
*hAT-Charlie*	100	108	99	58	72	76	79	76	78	101	847	317
*hAT-Tip100*	19	17	18	10	19	16	16	13	13	15	156	63
*TcMar-Tigger*	44	51	49	28	47	49	57	36	58	59	478	221
*TcMar-Mariner*	7	8	8	2	6	7	8	7	6	15	74	34
Others	17	18	17	56	12	15	17	11	15	17	195	113
**All Retro-DNAs**	**187**	**202**	**191**	**154**	**156**	**163**	**177**	**143**	**170**	**207**	**1,750**	**748**

By sequence length, these 748 (after removing orthologous redundancy ( Table 2)) retro-DNAs averaged at 209 bp (±190 bp) in length, representing in all cases only part of the corresponding family consensus sequences (averaging at 21%) ( Table 3). While the consensus sequences for DNA transposon families differ in length significantly, ranging from 380 bp for *TcMar-Mariner* to 1,506 bp for *hAT-Tip100*, the average length of retro-DNAs seems to be relatively more consistent across the families, ranging from 122 bp for *TcMar-Mariner* to 251 bp for *TcMar-Tigger.* Nevertheless, in general, the retro-DNAs from the longer families do have a longer average length ( *e.g.* *hAT-Tip100*) than those from the shorter families, but at lower proportions of their consensus sequences than those with shorter consensus sequences ( *e.g. TcMar-Mariner*) ( Table 3), suggesting more chance for 5′ truncation than the short ones as expected. This may be considered as another characteristics of the TPRT-driven retrotransposition.

**Table 3.** The composition of retro-DNA by family and the size information.

DNA transposon Family	copy number	% of all retro-DNAs	Average size (bp)	Std (bp)	Average consensus length (bp)	% of consensus
*hAT-Charlie*	317	42.4	190	110	515	37
*TcMar-Tigger*	221	29.5	251	256	1,162	22
*hAT-Tip100*	63	8.4	200	209	1,506	13
*TcMar-Mariner*	34	4.5	122	115	380	32
Other	113	15.1	210	200	1,053	20
**Total**	**748**	100	**209**	**190**	**923**	**21**

**Figure 4.**
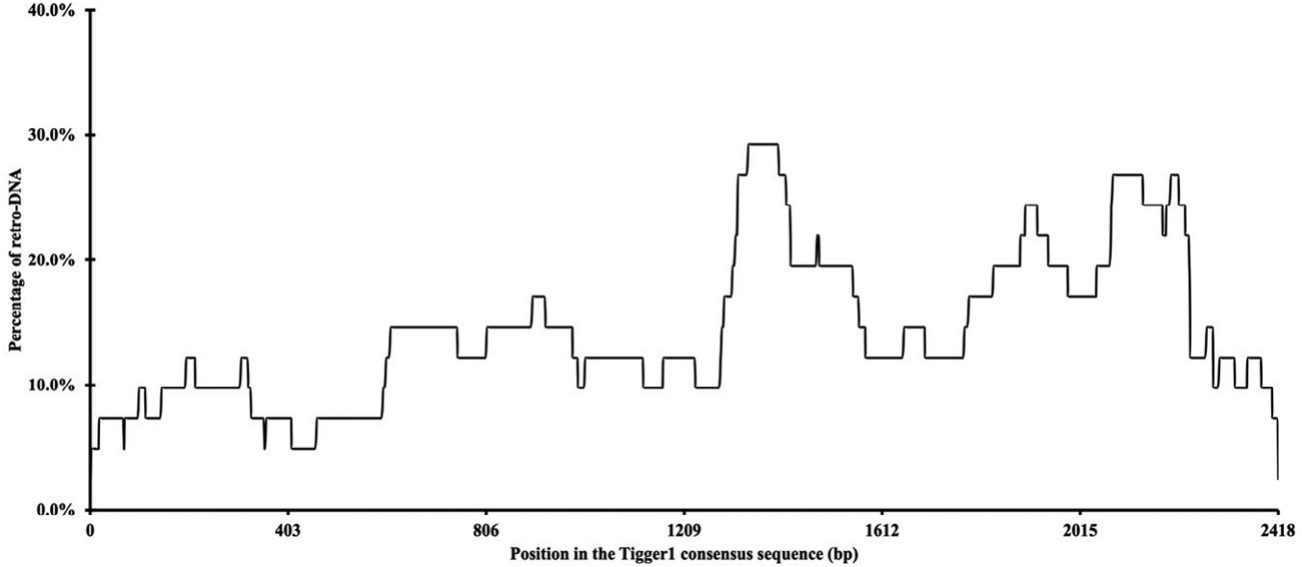
A frequency of the *Tigger1* subfamily DNA transposon consensus sequence used for retro-DNA sequences. The plot is based on the data for a total of 41 non-redundant retro-DNA entries from the Tigger1 subfamily.

Additionally, we examined whether there were any hotspots in these DNA transposon sequences as the source sequences of these retro-DNAs. By using the retro-DNA entries from the *Tigger1* DNA transposon subfamily, which is the largest subfamily containing 41 non-redundant retro-DNAs, we generated a frequency plot to show the usage of the consensus sequences by the retro-DNAs. As illustrated in Figure 4, while all regions of the consensus sequence were covered by the 41 retro-DNAs, the frequency varied substantially from 2.4% to 29.3%, showing that a few regions of the consensus sequence ( *e.g.* ~1310-1440 bp and ~1840-2240 bp) were used more frequently than the rest of the regions. The overall lower representation for the 5′ end of the consensus may suggest there might be a preference for the plus strand for using as retro-DNA at least for this family and presence of 5′ truncation.

**Figure 5.**
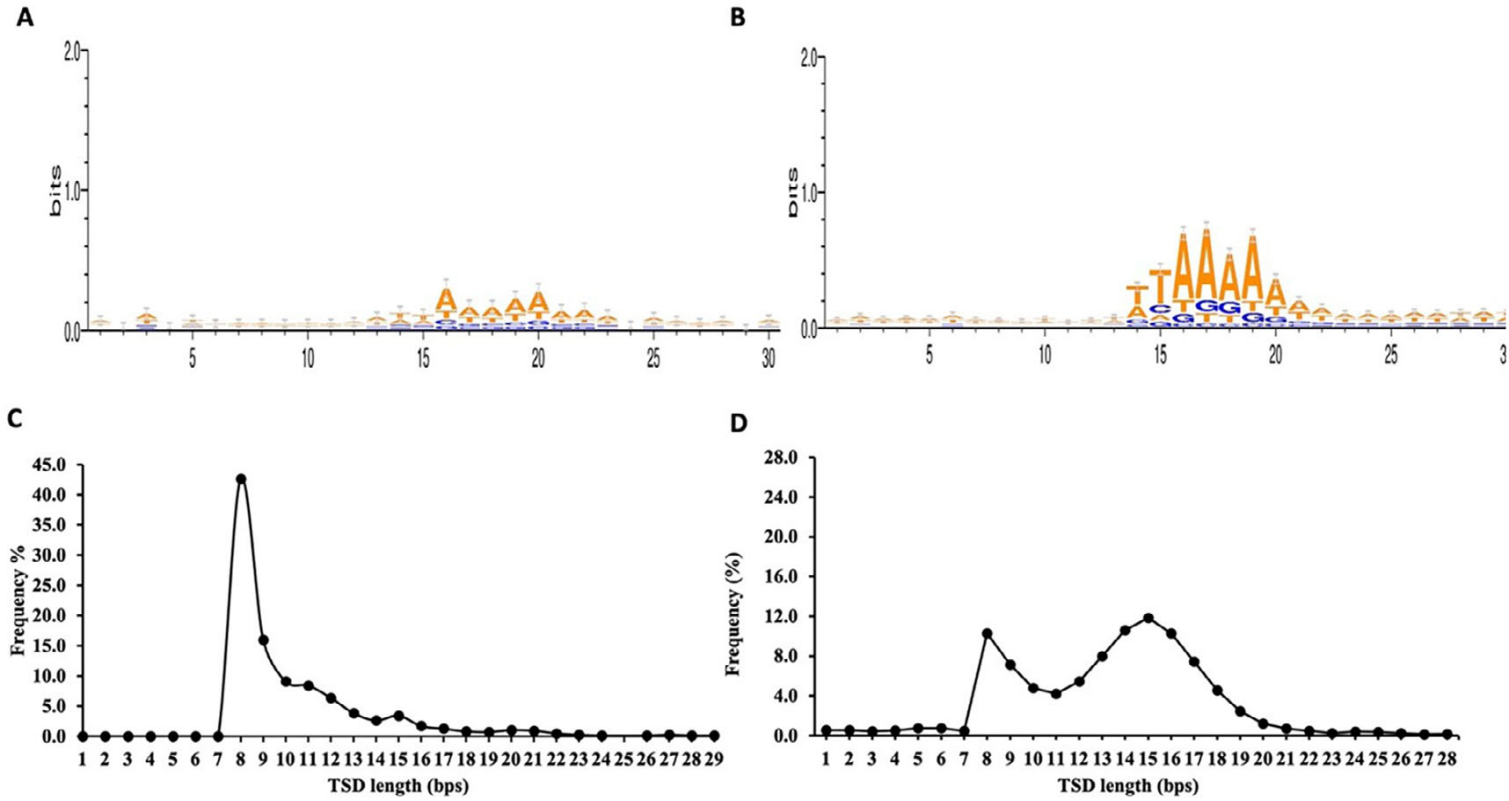
Sequence motifs of pre-integration sites and target site duplications (TSDs) length distribution pattern for retro-DNAs. A. Sequence motif logos for retro-DNAs at the integration sites. B. Sequence motif logos for human-specific L1s at the integration sites, adopted from authors’ publication. ^3^ C. A line plot showing the distribution of TSD length for retro-DNAs. D. A line plot showing the distribution of TSD length for human-specific L1s, adopted from authors’ publication. ^3^

From the total 748 non-redundant retro-DNAs, we identified 176 entries carrying a potential polyA tail (Table S2). We speculate that the relatively low percentage (23.5%) of entries with a polyA tail might be partially due to polyA sequences being more prone to sequence divergence from random post-insertion mutations for their homopolymer nature. The complete list of the 748 non-redundant retro-DNA entries with their genomic coordinates in all applicable genomes is provided in Supplementary File 1. For these retro-DNA insertion events, we further examined the sequence motifs at the insertion sites and the TSD length distribution pattern. As shown in Figure 5A, a sequence motif of ‘TT/AAAA’, same as the motif for *Alu*s, L1s, and SVAs ( Figure 5B), ^32^^,^^36^^,^^41^ was observed, despite the signal being much weaker. This, nevertheless, serves as a strong indication of their use of the L1-based TPRT machinery. ^33^^,^^34^ As further support, the TSD length distribution peaked at 8 bp ( Figure 5C), similar to the second peak seen for the TSDs of human specific L1s, despite missing the major peak at 15 bp observed for the latter ( Figure 5D). ^36^

### The species- and lineage specific pattern of retro-DNAs

**Figure 6.**
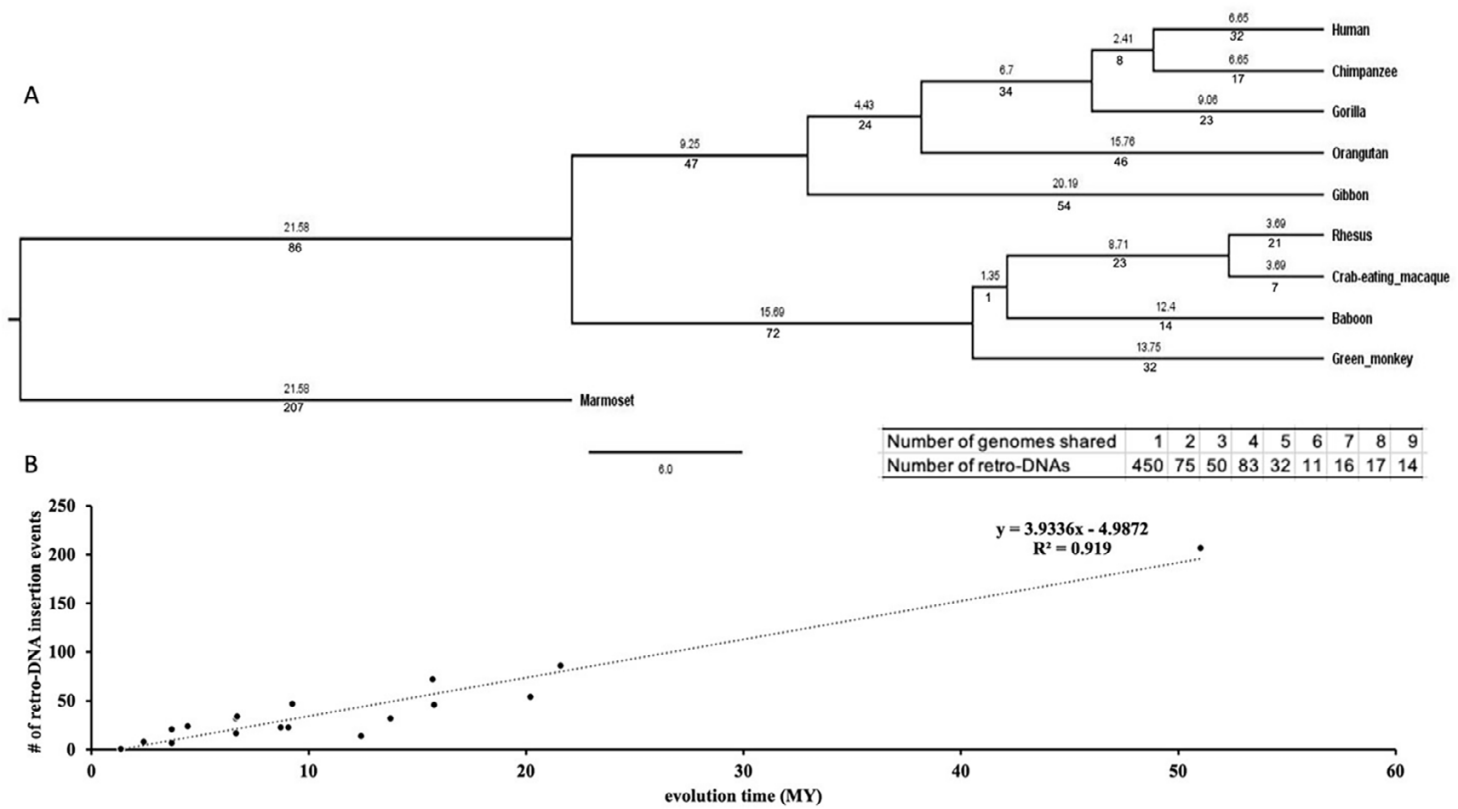
The evolutionary timeline of the retro-DNA insertions during the evolution of the ten primate genomes. A. A rooted phylogenetic tree of the ten primate genomes from the TimeTree database ( http://www.timetree.org/). The numeric values below each branch represent the number of retro-DNA insertion events happened during the corresponding period of primate evolution. The numeric value above each branch represents the millions of years (Mya) for that branch. The evolutionary time for marmoset has been manually corrected from 21.58 MY to 51.02 MY for the correlation analysis in panel B. The table insert below the tree shows the distribution of the retro-DNAs by the degree of conservation among the genomes as measured by the number of genomes owning a retro-DNA. B. A scatter plot between the number of retro-DNA insertion events and their evolutionary age based on the data in panel A. The trend line shows that the number of retro-DNA insertion events is positively correlated with the relative evolutionary distance (R ^2^ = 0.919).

We examined the evolutionary timeline of the retro-DNA insertion events by mapping them onto a phylogenetic tree of these primates based on the data in the TimeTree database. ^43^ As shown in Figure 6A (the insert), 450 (60.2%) of these retro-DNAs appeared to be species-specific for being uniquely present in only one genome, while another 295 (39.4%) were found in multiple genomes in a clear lineage-specific pattern. On average, a retro-DNA was shared by two genomes, implying an average age older than the species-specific MEs (unique to one species) reported in our earlier study. ^3^ As shown in Figure 6B, the number of retro-DNA insertional events appears to show a positive linear correlation with the relative evolutionary ages of the species and lineages (R ^2^ = 0.5463), suggesting that these retro-DNA insertional events occurred at a low but relatively consistent rate during primate evolution.

**Figure 7.**
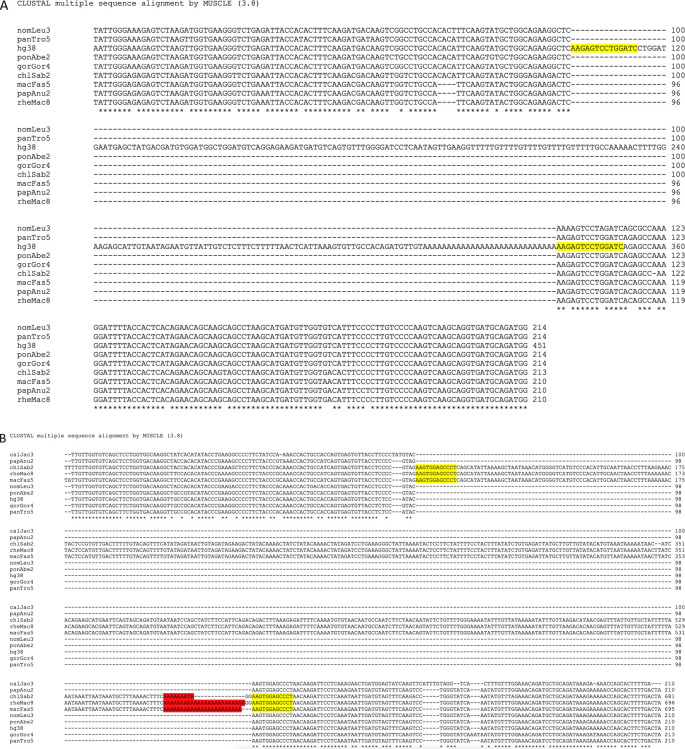
Multiple sequence alignment and phylogenetic analysis of retro-DNAs. A. Multiple sequence alignment for a retro-DNA located in the human genome (hg38_chr4:146335052-146335253, the same entry in Figure 1A) and the corresponding pre-integration sequences from the other eight primate genomes. The pre-integration sequence from the marmoset genome was not identified likely due to the high level of sequence divergence. B. Multiple sequence alignment for the sequences of a retro-DNA shared among green monkey, crab-eating macaque and rhesus genomes (chlSab2_chr8:30005081-30005527, macFas5_chr8:32527581-32528029, and rheMac8_chr8:31992158-31992606) with the flanking sequences, along with their orthologous pre-integration sequences from 7 other primate genomes. The red highlights indicate possible polyA tails with variable lengths across genomes, while the yellow highlights show the observed target site duplications (TSDs).

The example shown in Figure 2A serves as a very clear case of species-specific retro-DNA. As shown in the multiple sequence alignments with its orthologous sequences including its flanking sequences from other eight primate genomes (not locatable in marmoset genome), this *Tigger7* element was absent from the orthologous sites of all non-human primate genomes ( Figure 7A), confirming it as an authentic human-specific retro-DNA. On the contrary, the example shown in Figure 2B is shown to be a retro-DNA insertion event shared among three of the four monkey species and absent in the orthologous regions of the remaining seven primate genomes, thus likely as a lineage-specific retro-DNA ( Figure 7B). Furthermore, it appears that this retro-DNA sequence in these three genomes had been subject to mutation in the polyA tails shown as having variable lengths, agreeing with its relatively older age as a lineage-specific retro-DNA and the higher rate of mutation in the polyA region. Similarly, the example shown in Figure 2D represents an ape lineage-specific retro-DNA for its presence in all ape genomes but absent in all non-ape genomes examined.

### The genome distribution patterns of retro-DNAs and their parent sites in gene context and expression

To assess the potential functional impact of these retro-DNAs, we examined their gene context based on the Ensembl gene annotation for these genomes. ^42^ A total of 698 retro-DNAs, representing ~40% of the 1,750 retro-DNAs were located within the genic regions and promoter regions for 734 transcripts from 414 unique genes ( Table 4 and Table S3). Majority of these retro-DNAs were located within the intron regions (699/734 transcripts), while 27 entries were inserted into promoter regions and untranslated regions. The presence of these retro-DNAs in the genic regions provides them the potential to impact gene regulation or splicing.

**Table 4.** The numbers of retro-DNAs located in the genic regions in the 10 primate genomes.

Genic region ^*^	Human	Chimpanzee	Gorilla	Orangutan	Gibbon	Crab-eating macaque	Rhesus	Baboon	Green monkey	Marmoset	Total
NR	4	1	1	1	0	0	1	0	0	0	8
Promoter	9	5	2	1	0	1	1	0	0	3	22
5′ UTR	0	1	0	0	0	1	0	0	0	0	2
3′ UTR	1	0	0	0	0	0	0	0	0	2	3
Intron	114	78	70	60	61	62	67	42	53	92	699
**Total**	**128**	**85**	**73**	**62**	**61**	**64**	**69**	**42**	**53**	**97**	**734**
**Total (nr)**	**109**	**82**	**70**	**60**	**61**	**63**	**67**	**42**	**53**	**91**	**698**

^*^ , NR: non-coding RNA; UTR: untranslated region

**Figure 8.**
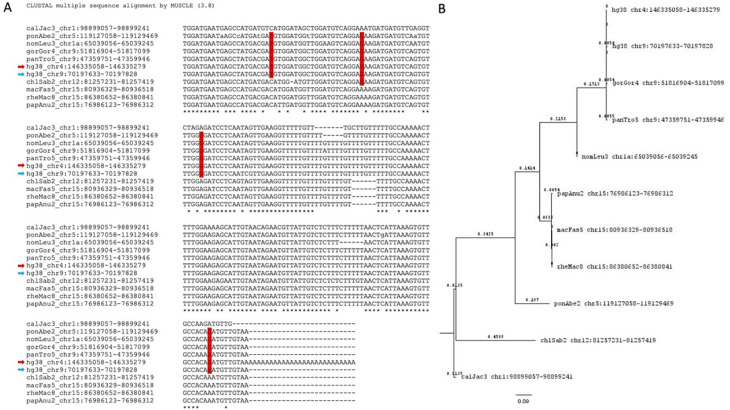
Sequence alignment and phylogenetic analysis of a human retro-DNA, its parent copy in the same genome, and its orthologous copies in other genomes. A. Multiple sequence alignment for a retro-DNA in the human genome (hg38_chr4:146335052-146335253) and its parent copy (hg38_chr9:70197633-70197828, showing only the sequence aligned with the retro-DNA) plus the orthologous sequences of the parent copy from the other 9 non-human primate genomes. The red arrow indicates the retro-DNA entry, while the blue arrow indicates the parent copy. Shared SNPs in red vertical boxes are seen among members of the *Hominidae* group. B. Phylogenetic analysis of the 11 DNA sequences from the 10 primate genomes shown in A using the Maximum Likelihood method and Tamura-Nei model. ^62^ The bootstrapped consensus tree inferred from 500 replicates ^63^ is used to represent the evolutionary history of the taxa involved. Branches corresponding to partitions reproduced in less than 50% bootstrap replicates were collapsed. The percentage of replicating trees in which the associated taxa clustered together in the bootstrap test (500 replicates) are shown next to the branches. ^63^ Initial tree(s) for the heuristic search were obtained automatically by applying Neighbor-Joining and BioNJ algorithms to a matrix of pairwise distances estimated using the Maximum Composite Likelihood (MCL) approach followed by selecting the topology with superior likelihood value in logarithmic scale. This analysis involved 11 DNA sequences with a total of 222 positions in the final dataset.

Further, we identified the potential parent sites for these retro-DNAs by performing a sequence similarity search using their sequences to query the corresponding genome sequences. For each retro-DNA, the best non-self-match was selected as its potential parent site. An example of such a parent-child relationship is shown in Figure 8, in which a human-specific retro-DNA event on chromosome 4 is shown to be a child to a much longer Tigger7 (1882 bp) on chromosome 9, which has orthologous copies in other primate genomes, indicating a much older age of the latter and its validity as a parent copy for the former. As shown in Table S4, we identified a total of 715 potential parent sites for the 1,750 retro-DNA entries (or 325 entries for the 748 retro-DNAs after removing the redundancy across species). The failure in finding the parent copies for the remaining entries could be due to the loss of the parent copy from genomic rearrangements or due to incomplete sequence coverage of the genomes. Like for the retro-DNAs, we examined the gene context for these potential parent sites, and as shown in Table S5, 351 (49.1%) of these redundant potential retro-DNA parent sites locate to 410 different genic regions for 371 unique genes. In these cases, the transcripts of these potential parent sites, likely as part of the transcripts or splicing by-products ( *e.g.*, excised intron sequences) of their host genes, might have had the chance to be captured by the L1 TPRT machinery to generate retro-DNAs as in the case of processed pseudogenes/retro-genes. The ratio of genic entries (49.1%) was higher for the parent sites than that for retro-DNAs (~40%), and the implication of this is discussed later.

We also examined the expression level of retro-DNAs and their potential parent sites using RNA-seq data from the Non-Human Primate Reference TRanscriptome (NHPRTR) dataset ^44^ and two other studies ^45^^,^^46^ to see if any of these entries had any transcriptional activity in the present-day primate genomes. For this, we collected a total of 21 transcriptomes for seven primates, excluding orangutan, gibbon, and marmoset, for which no transcriptome data was available at the time of our analysis. To minimize false positives due to the high sequence similarity among ME members in the same family, we included only the reads with a perfect match to the retro-DNAs or their parent site regions and with each read used only once in calculating the expression level. However, we believe that this process has inevitably introduced a certain level of false negatives in the results due to sequence polymorphisms and, therefore, may have led to an underestimation of the retro-DNAs and parent sites’ expression levels. As seen in Tables 5 and S6, 966 loci from the 1,750 retro-DNA and 715 parent sites in these seven primate genomes were shown to have a certain level of expression ranging in fragments per kilobase of transcript per million reads (fpkm) value from 0.0003 to 27.3.

**Table 5.** The numbers of expressed retro-DNAs and parent sites in 21 primate transcriptomes.

Species	# of RNA-seq sets	retro-DNAs			parent sites		
		# of entries	# of expressed	%	# of entries	# of expressed	%
Human	6	187	93	49.7	98	57	58.2
Chimpanzee	2	202	99	49.0	101	67	66.3
Gorilla	1	191	55	28.8	99	42	42.4
Rhesus	4	177	97	54.8	64	46	71.9
Crab-eating macaque	4	163	115	70.6	63	55	87.3
Baboon	2	143	68	47.6	53	34	64.2
Green monky	2	170	90	52.9	62	48	77.4
Total	19	1063	527	49.6	478	301	63.0

**Figure 9.**
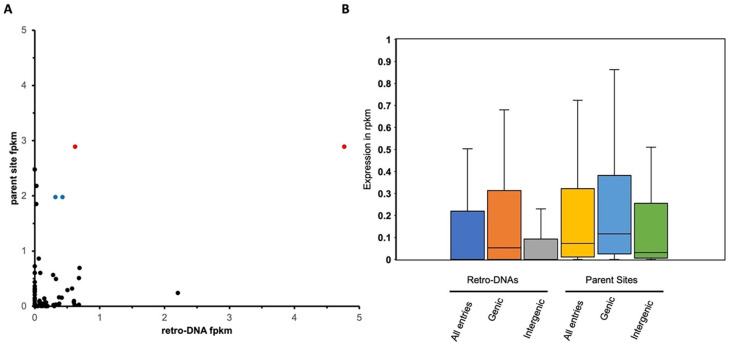
The expression level of retro-DNAs and their parent sites in three human testis transcriptomes. A. A scatter plot based on 66 retro-DNA/parent site pairs which show a certain level of expression (fpkm > 0) for the retro-DNA and/or parent site. The two data points in red with the same value for the parent but different values for the retro-DNA copies point to the same parent copy in human at hg38:chr5:1570263-1570333, and the two data points in blue point to the same parent copy in human at chr5:259441-262665. B. Box plots showing the expression levels of the 66 retro-DNAs and parent sites divided into genic and intergenic groups. Expression data was based on the average fpkm value in the three human testis transcriptomes.

We further investigated the relationship between retro-DNAs and their parent sites based on their expression levels. Specifically, three human testis transcriptome samples (SRR2040581, SRR2040582, SRR2040583) retrieved from the NCBI SRA ( Sequence Read Archive)) were used to analyze the expression level of the retro-DNA/parent site pairs. As shown in Figure 9A, a total of 66 retro-DNA/parent site pairs were shown to have a certain level of expression (fpkm > 0) for either the retro-DNA or the parent site among the three human testis samples. Notably, among these 66 retro-DNA/parent site pairs, 57 (86.4%) parent sites were shown to be expressed (fpkm > 0) compared to only 42 (63.6%) expressed retro-DNAs (Table S4 and S6, Figure 9A). This difference might indicate that the generation of a retro-DNA requires the expression of its parent site, while a retro-DNA itself may not be expressive depending on its landing location. Therefore, a higher ratio of transcriptionally active sites may be expected for the parent sites than for the progenies (retro-DNAs). More interestingly, the two parent sites responsible for multiple retro-DNA entries were shown to have the highest levels of expression among the parent sites ( Figure 9A). This may suggest that the expression level of the parent sites is positively correlated to their potential in generating retro-DNAs. Furthermore, the ongoing expression of the parent sites in the current genomes suggests that they have the potential to generate more retro-DNAs in the future.

We also examined and compared the expression levels of retro-DNAs and their parent sites among gene context-based groups in the three human testis transcriptomes. One critical reason for including testis here is to ensure that these retro-DNAs are expressed in germline cells for generating transmissible daughter copies. As shown in Figure 9B, the average fpkm values of the parent sites were always higher than that of the retro-DNA entries as a whole group or divided into genic and intergenic regions. In addition, the entries located within genic regions showed higher expression than the ones located outside the genic regions for both retro-DNAs and the parent sites ( Figure 9B), suggesting that entries located in the genic regions may have more opportunities to be expressed passively as part of the host gene expression. This difference is larger for retro-DNAs than for the parent sites, likely because parent sites had to be expressed regardless of their position in order to be able to generate new copies. None of these differences are statistically significant, likely due to the small sample size.

## Discussion

### Retro-DNAs as retrotransposons derived from DNA transposons

In this study, we focused on a small number of species-specific DNA transposons identified in primate genomes using a computational comparative genomics pipeline previously established for analyzing species-specific retrotransposons in the human genome and seven other genomes. ^3^^,^^36^ Unlike for retrotransposons, for which the ongoing activity during evolution and in the current genomes of primates, as well as their contribution to the lineage- and species-specific MEs, have been well established, ^3^^,^^32^^,^^47^ similar research for DNA transposons in primate genomes remains very scarce. As a matter of fact, as at time of writing, no report of species-specific DNA transposons in these primate genomes has been documented, likely due to lack of effort, as DNA transposons are thought to have become inactive in primate genomes about 37 Mya. ^17^^,^^19^

In trying to understand the mechanism underlying the mysterious species-specific DNA transposon insertions identified in our comparative genome analysis, we spotted a few interesting entries as exemplified by the case shown in Figure 2A, which manifests the characteristics of non-LTR retrotransposons by having longer TSDs and presence of a polyA tail, while lacking TIRs, the hallmark of new DNA transposon insertions. The remaining cases shown in Figure 2 have the same non-LTR features but do not necessarily have a typical polyA tail. For their non-LTR retrotransposon characteristics, we named them “retro-DNA” for being retrotransposons derived from DNA transposons. We then performed a systematic analysis to look for more of such “retro-DNA” cases.

For this, we expanded our search from the strict species-specific DNA retrotransposons, which are defined as those present in only one of the primate genomes, ^3^^,^^36^ to da-DNAs, which are defined as diallelic DNA transposons with the insertion allele and its pre-integration allele ( *i.e.*, the orthologous region without the DNA transposon) both present in at least one of the ten genomes we included. We obtained a total of 271,085 da-DNAs, and from these we then specifically searched for retro-DNA cases, which have long TSDs (≥8bp) and the absences of the TIRs using a protocol shown in Figure 3. This led to the identification of 1,750 of retro-DNA cases, which represent 748 unique events, covering all ten primate genomes with over half being species-specific and the rest being lineage-specific covering different lineages in this group of primates ( Figure 6A). Our results indicate that the generation of retro-DNAs has occurred in all ten primate genomes included in our analysis and at a wide-spectrum of evolutionary time with an approximately constant rate ( Figure 6). Furthermore, these retro-DNAs are not limited to a single subfamily, but rather cover all major DNA transposon families, suggesting that the existence of such “retro-DNAs” is the product of a consistent and common process actioning in primate evolution.

### The likely mechanism underlying the generation of retro-DNAs

Several lines of evidence from our results guided us to propose that these retro-DNAs were the products of the L1-based TPRT machinery, similar to the known non-autonomous non-LTR retrotransposons, *i.e.*, SINEs, SVAs and processed pseudogenes. ^9^^,^^33^^–^^36^ The major pieces of evidence include the lack of TIRs and the presence of the TPRT insertion site sequence motif and long TSDs. As seen in Figure 5A, the integration sites of the 748 retro-DNAs display, although at a much weaker signal, is the exact same core sequence motif of “TT/AAAA”, found for non-LTR retrotransposons in the human genome ( Figure 5B). ^34^^,^^36^^,^^41^ The TSDs for these retro-DNAs show a dominant peak at 8bp ( Figure 5C), which is much longer than that of TSDs typically found for DNA transposons (2 bp) and is similar to the secondary peak of TSD length observed for the human-specific L1s ^36^ ( Figure 5D). Furthermore, the presence of parent sites in the same genome for a significant proportion of the retro-DNAs (325/748 or 43.5%) indicates their use of a “copy-and-paste” rather than the “cut-and-paste” mechanism used by canonical DNA transposons. The presence of a polyA tail in many (176/748 or 23.5%) of these retro-DNAs and the apparent occurrences of the 5′ truncation suggested by data in Table 3, Figure 4 provide additional support for their use of the L1-based TPRT mechanism.

It is worth noting that, as described above, while there is sufficient similarity in sequence features between these retro-DNAs and the known non-LTR retrotransposons for treating these retro-DNAs as non-LTR retrotransposons, several unique aspects are also evident. These include the missing of the major TSD length peak at 15 bp observed for other non-LTR retrotransposons, the low percentage of entries with a polyA tail, and the weaker signal of the sequence motif, “TT/AAAA”, at the integration sites. All of these unique characteristics might be attributed to the relatively older average age of these retro-DNAs as indicated by the relatively high percentage (298/748 or ~40%) for being lineage-specific ( Figure 6A) compared to the non-LTR retrotransposons used in most previous studies for analysis of integration site sequence motifs. ^9^^,^^33^^–^^36^ In other words, the older age of the retro-DNAs leads to higher sequence divergence, which in turn lowers the sensitivity for detecting all of these sequence features. An additional reason for the weaker signal in the insertion site sequence motif for the retro-DNAs could be due to the small sample size. It is also possible that these unique characteristics may suggest that some differences in the detailed retrotransposition process of these DNA transposons, likely with regard to the interaction between the retro-DNA transcripts and the ORF1 and ORF2 proteins, may exist between the retro-DNAs and the canonical non-LTR retrotransposons. One known example for this is that Alu transposition does not seem to require ORF1p unlike for L1s. ^32^^,^^48^^,^^49^

It is also worth pointing out that in addition to the well-known types of non-autonomous non-LTRs transposed by the TPRT machinery, including SINEs, SVAs, and retro-genes, evidence suggests that some copies of the LTR-retrotransposon subfamily, HERV-W, might have also been transposed by this mechanism. ^50^^,^^51^ However, these HERV-W sequences are part of retrotransposons and can continue to be transposed using their canonical retrotransposition mechanism. For this reason, we would like to argue that our identification of retro-DNAs is unique and significant in the sense that they represent DNA transposons, which would not be able to transpose anymore in the primate genomes, since their canonical mechanism is no longer active. Overall, the research from this study and others clearly suggests that the L1-based TPRT machinery may be able to transpose a much wider variety of genomic sequences than what are currently known.

### The relative retro-DNA activity during primate evolution

In comparison with the other types of non-autonomous non-LTR retrotransposons, including Alus, SVAs, and processed pseudogenes, in primate genomes, ^2^^,^^3^^,^^32^^,^^52^ the number of retro-DNAs per genome was much lower, averaging at < 200 per genome (Table S2). This number was even substantially lower than that of processed pseudogenes, which represent the smallest class of non-LTR retrotransposons with 10,190 copies in the human genome. ^53^ We reason that the very small copy number of retro-DNAs may primarily attribute to one factor, *i.e.*, the lack of intrinsic internal promoters to drive their own transcription, leading to an overall low level of their transcripts available for retrotransposition. In contrast, retrotransposons carry their intrinsic promoters required for their canonical propagation mechanisms, while a promoter is not required for the canonical DNA transposon activity. This is in agreement with the observation that there is no clear hotspot in the DNA transposon consensus sequences used in generating retro-DNAs, as shown in Figure 4 for Tigger1. Should there be internal promoters driving the transcription, we would expect to observe one or more clear dominant peaks in the frequency of the regions used for retro-DNAs correlated with the location of the internal promoter(s). Without the ability to drive their own transcription, the only way for DNA transposons to get transcribed is to get transcribed as a part of the host gene transcripts. If this is how retro-DNAs were generated, then we would expect to see a high percentage of retro-DNAs having their parent sites located in the genic regions, more specifically in the transcribed regions, *i.e.* exon and intron regions. By examining the gene context, 351 of the 715 parent sites (49.0%) for the retro-DNAs were found to locate in 371 unique genes/transcripts in the ten primate genomes. This ratio was higher than that for all DNA transposons in the genic regions (39%, detailed data not shown) as the expected for random distribution and for that of the retro-DNAs (40% in genic sites including promoters) ( Table 4 and Table S5), thus supporting the role of passive expression for the parent sites in generating these retro-DNAs.

By the same rationale, we would expect that on average the parent sites should have a higher expression level than retro-DNAs since the parent sites were selected to be biased for this by locating in the genic regions, while the location of the retro-DNAs is more or less random, leading to a relatively lower proportion in genic regions than the parent sites as shown in our data (40% verse 49%) ( Table 4, Table S5). This is supported by the expression data showing that among the 66 retro-DNA/parent site pairs, 57 pairs have parent sites with a fpkm > 0 compared to only 42 expressed entries for retro-DNAs ( Figure 9A). Additionally, we identified two parent sites, which are the only sites potentially responsible for generating multiple retro-DNA entries, and they showed the highest levels of expression among the parent sites ( Figure 9A). By comparing the expression levels of all parent sites with that of retro-DNAs in the human genome, we can see an overall higher expression for the parent sites ( Figure 9B), and this is also true when comparing between the sites in the genic and intergenic regions ( Figure 9B). Furthermore, the expression level of parent sites in the genic regions is much higher than their counterparts in the intergenic regions as expected ( Figure 9B). Another possible factor contributing to the extremely small number of retro-DNAs might be that the sequences of these DNA transposons are much less optimal for TPRT-based retrotransposition than the canonical types of retrotransposons.

The use of the 10 primate genomes, representing several lineages with a large span in primate evolution, allowed us to examine whether there is any positive correlation between the length of evolutionary span and the number of retro-DNA insertional events. As shown in Figure 6B, a moderate positive correlation between the two is observed (R ^2^ = 0.5463), suggesting that the generation of retro-DNAs is relatively steady during the evolution of this group of primates. Furthermore, the observation that many of the retro-DNA parent sites, as well as 966 of the 1773 (~54.5%) retro-DNAs show certain levels of expression in the seven primate transcriptomes ( Table 5 and Table S6), suggests the possibility of ongoing retro-DNA generation from the parent sites and perhaps also from some retro-DNAs.

### Conclusions and future perspectives

In this study, through a comparative genomic analysis of 10 primates, we report the first identification of “retro-DNAs” for being non-LTR retrotransposons derived from DNA transposon sequences. This work is significant, as the generation of these retro-DNAs serves to propagate DNA transposon sequences in the absence of the canonical DNA transposon activity in primate genomes and the process involves two fundamentally different ME classes. Despite being very small in number, they do contribute to the genetic diversity among primate species along with other MEs, and our data seem to suggest that at least some of them have the capability to serve as a parent copy to further propagate, differentiating them from other elements that are passively retrotransposed by the L1 machinery, such as processed pseudogenes. ^54^ Furthermore, the discovery of these retro-DNAs suggests that the L1-based TRPT machinery may have been used by more diverse types of RNA transcripts than what we currently know. Interesting follow-up work ought to include the verification of the retrotransposition activity of these retro-DNAs and their parent sites using *in vitro* and in *vivo* assays ^55^ and extension of the similar analysis to other types of expressive DNA sequences, such as non-coding RNA genes. Should their intrinsic capacity of retrotransposition as in the case of Alus and SVAs be experimentally verified, we could then classify these retro-DNAs as a new type of non-LTR retrotransposons beyond the current LINE, SINE, and SVA. In addition, research into the mechanisms underlying the remaining majority of the diallelic DNA transposons would also be very interesting and valuable.

## Methods

### Sources of primate genome sequences

In this study, we chose to use 10 primate genomes including human, among which eight genomes were included in our previous study for identifying species-specific MEs in primates. ^3^ These 10 primate species include human (GRCh38/UCSC hg38), chimpanzee (May 2016, CSAC Pan_troglodytes-3.0/panTro5), gorilla (Dec 2014, NCBI project 31265/gorGor4.1), orangutan (July 2007, WUSTL version Pongo_albelii-2.0.2/ponAbe2), gibbon (Oct. 2012 GGSC Nleu3.0/nomLeu3.0), green monkey (Mar. 2014 VGC Chlorocebus_sabeus-1.1/chlSab2), crab-eating macaque (Jun. 2013 WashU Macaca_fascicularis_5.0/macFas5), rhesus monkey (November 2015 BCM Mmul_8.0.1/rheMac8), baboon (Anubis) (March 2012 Baylor Panu_2.0/papAnu2), and marmoset (March 2009 WUGSC 3.2/calJac3). The marmoset genome was added to expand the evolutionary span, also serving as an outgroup for the other nine genomes from the ape and monkey groups, while the gibbon genome was added to increase the coverage and evolutionary span of the ape group. All genome sequences in fasta format and the RepeatMasker annotation files were downloaded from the UCSC genome website onto our local high performance computing servers for in-house analyses. We have used the most recent genome versions available on the UCSC genome browser website at the time of analysis in all cases except for gorilla, for which there is a newer version (March 2016, GSMRT3/gorGor5) available but not scaffolded into chromosomes, making it inadequate for our analysis.

### LiftOver overchain file generation

A total of 90 liftOver chain files were needed for all possible pair-wise comparisons of the 10 genomes used in this study. These files contain the information linking the orthologous positions in a pair of genomes based on lastZ alignment. ^56^ A total of 22 of these were available and downloaded from the UCSC genome website, and another 34 liftOver chain files were generated using a modified version of UCSC pipeline RunLastzChain from a previous study. ^3^ The remaining 36 liftOver chain files were newly generated for this study using the same pipeline.

### Identification of DNA transposons with diallelic status in the ten primate genomes

**Pre-processing of DNA transposons:** The starting list of DNA transposons in each primate genome was obtained based on the RepeatMasker ME annotation data from the UCSC website. As previously described, we performed a pre-processing to integrate the ME fragments annotated by RepeatMasker back to ME sequences representing the original transposition events. ^36^

**Identification of DNA transposons with diallelic status:** We modified a previously reported comparative genomics bioinformatics pipeline ^36^ to identify da-DNAs that have the presence of both the insertion and pre-integration alleles in at least one of the 10 primate genomes. Briefly, this pipeline uses a robust multi-way computational comparative genomic approach to determine the presence/absence status of DNA transposons among a group of genomes by using both the whole chromosome alignment-based liftOver tool and the local sequence alignment-based BLAT tool. ^57^^,^^58^ The sequence of a DNA transposon at the insertion site and its two flanking regions in a genome were compared to the sequences of the orthologous regions available in all other genomes. If a DNA transposon is absent from the orthologous regions of any of the other nine genomes not due to the existence of a sequence gap ( *i.e.* just missing the insertion), it is selected as a potential candidate of da-DNA subject to further analyses.

### Identification of retro-DNAs

**Identification of TSDs and TIRs:** For the candidate entries from the previous step, using in-house PERL scripts as described previously, ^36^ we performed identification of the TSDs. Additionally, we modified our scripts to identify the TIRs, the hallmark of all cut-and-paste transposons except for *Helitrons.* ^17^ da-DNA entries without identifiable TSDs or TSD length < 8 bp, as well as entries with identifiable TIRs, were excluded from further analysis. The 8 bp TSD length cutoff was chosen based on our observation for human-specific retrotransposons that 95% of identified TSDs are at least 8 bp long. ^36^ Additionally, we used MiteFinderII, a tool designed to identify miniature inverted-repeat transposable elements, ^59^ to verify that none of our candidate entries contain TIRs.

**Filtering against retrotransposon transductions:** To ensure the presence of a DNA transposon was a result of active transposition, rather than a passive result of other processes, *e.g.*, retrotransposition-mediated transductions, we mapped the candidate entries against the known retrotransposons in the ten primate genomes based on their genomic positions. Specifically, the sequences of candidates from the previous step were mapped back onto the host genome using BLAT, followed by removing all entries located within 50 bps to a retrotransposon (excluding entries inserted into a retrotransposon), because such entries could be a result of retrotransposition-mediated transduction. All entries left at this point were considered candidates of “retro-DNAs” for being retrotransposons derived from DNA transposon sequences but lacking TIRs and having TSD at 8 bp or longer.

**Identification of polyA tail:** For each candidate retro-DNA, we retrieved the 10 bp sequence from the 3’ end of the positive-strand (by the DNA transposon consensus sequence). If the sequence contains six or more “A”, the entry is considered to have a polyA tail.

### Clustering retro-DNAs to identify unique retro-DNA events

The retro-DNA candidates identified from the last step in the 10 primate genomes were subject to a round of “all-against-all” sequence similarity search using BLAT with the sequences of the retro-DNAs plus the 100 bp of the flanking region on each side. Entries with 95% or higher sequence similarity across the entirety of the sequences including the flanking sequences were identified as one orthologous cluster, representing one retro-DNA insertion event during the evolution of these primates.

### Estimating the timeline for retro-DNA insertions

An organismal phylogenetic tree of the 10 primate genomes with the marmoset genome as the outgroup was obtained from the TimeTree database ^43^ and displayed using the Treeview program. ^60^ We then manually added the numbers of non-redundant retro-DNA entries onto the nodes and branches of this tree based on the presence of retro-DNAs in the specific genomes or lineages.

### Multiple sequence alignment of retro-DNA and parent sites

We performed multiple sequence alignment for a few selected retro-DNA entries, including their parent sites. For this, we first collected retro-DNA sequences including 100 bp on both flankings, as well as the orthologous sequences of the parent sites from the rest of primate genomes and performed multiple sequence alignment using the online version of MUltiple Sequence Comparison by Log-Expectation (MUSCLE) ^61^ from the European Bioinformatics Institute website. Phylogenetic analyses in some cases were performed using the Maximum Likelihood method and Tamura-Nei model ^62^ with bootstrapping ^63^ at 500 replications.

### Expression analysis of retro-DNAs and their parent copies

RNA sequencing (RNA-seq) data for the blood and the generic (mixed of twenty tissues) samples from chimpanzee, gorilla, crab-eating macaque, rhesus and baboon were retrieved from the Non-Human Primate Reference Transcriptome Resource (NHPRTR) ^44^ for expression analysis of the retro-DNAs and their parent copies. We also collected RNA-seq data for six human testis transcriptomes (three for blood and three for testis) ^46^ and two green monkey transcriptomes. ^45^^,^^64^ The detailed information regarding the NCBI SRA accession numbers and the associated species and tissues is available in Table S6. Tophat2 (version 2.1.1) was used to align the RNA-seq reads to the corresponding reference primate genomes. ^65^ Reads mapped to the retro-DNA/parent copies regions were retrieved in fasta format and aligned back to the reference genome using the NCBI blastn to ensure that each RNA-seq read was only assigned to only one genomic location with perfect match for use to calculate the fpkm values for each DNA transposon using an in-house Perl script.

### Facility and software for computational analysis

The data analysis and figure plotting were performed using a combination of Linux shell scripting, R, and Microsoft Excel. The computational analysis was mostly performed on Compute Canada high-performance computing facilities running CentOS Linux.

## Data Availability

BioStudies: The identification of retro-DNAs in primate genomes as DNA transposons mobilizing via retrotransposition, https://identifiers.org/biostudies:S-BSST1030. ^66^ Analysis code The customized perl and shell scripts used for identification of the reported retro-DNAs are available at https://github.com/pliang64/retro-DNAs . Archived analysis code at time of publication: https://doi.org/10.5281/zenodo.7682142. ^67^ License: GNU GPL-3.0
