# Supplementary material for: The identification of retro-DNAs in primate genomes as DNA transposons mobilizing via retrotransposition
Source: F1000Res. 2024 May 29;12:255. Originally published 2023 Mar 9. [Version 3] doi: 10.12688/f1000research.130043.3 (PMC11195612; doi:10.12688/f1000research.130043.3)
Supplement: Peer review history

# Reviewer report: Reviewer response for version 3

**DOI:** 10.5256/f1000research.166925.r284061

**Published:** 2024-06-18

**Authors:** Rene Massimiliano Marsano

**Affiliations:** 1 Università degli Studi di Bari "Aldo Moro", Bari, Italy

Thank you for addressing the issues I raised in my previous report.

Is the work clearly and accurately presented and does it cite the current literature?

Yes

If applicable, is the statistical analysis and its interpretation appropriate?

Yes

Are all the source data underlying the results available to ensure full reproducibility?

Yes

Is the study design appropriate and is the work technically sound?

Yes

Are the conclusions drawn adequately supported by the results?

Partly

Are sufficient details of methods and analysis provided to allow replication by others?

Yes

Reviewer Expertise:

transposable elements, genetics

I confirm that I have read this submission and believe that I have an appropriate level of expertise to confirm that it is of an acceptable scientific standard.

# Reviewer report: Reviewer response for version 3

**DOI:** 10.5256/f1000research.166925.r284062

**Published:** 2024-05-31

**Authors:** Shengjun Tan

**Affiliations:** 1 University of the Chinese Academy of Sciences, Beijing, Beijing, China

All of my concerns have been addressed. I am happy with current revision.

Is the work clearly and accurately presented and does it cite the current literature?

Yes

If applicable, is the statistical analysis and its interpretation appropriate?

Yes

Are all the source data underlying the results available to ensure full reproducibility?

Yes

Is the study design appropriate and is the work technically sound?

Yes

Are the conclusions drawn adequately supported by the results?

Yes

Are sufficient details of methods and analysis provided to allow replication by others?

Yes

Reviewer Expertise:

Transposon, TE-mediated gene duplication, genome evolution, new gene evolution

I confirm that I have read this submission and believe that I have an appropriate level of expertise to confirm that it is of an acceptable scientific standard.

# Response

**Published:** 2024-05-31

**Authors:** Ping Liang

**Affiliations:** Brock University, Canada

Dear Dr. Tan,

 Thank you very much for your prompt review of our revised manuscript and for your approval of the revisions. Your review and valuable inputs in improving the manuscript is sincerely appreciated.

 Best regards,

 Ping Liang

# Reviewer report: Reviewer response for version 2

**DOI:** 10.5256/f1000research.164971.r267657

**Published:** 2024-04-29

**Authors:** Shengjun Tan

**Affiliations:** 1 University of the Chinese Academy of Sciences, Beijing, Beijing, China

This study identifies retro-DNAs in primate genomes as a new type of mobile elements (MEs), derived from DNA transposons but mobilizing via retrotransposition. Several characteristics indicate that retro-DNAs use the L1-based target-primed reverse transcription (TPRT) mechanism for their mobilization. The presence of retro-DNAs in genic regions underscores their impact on gene function in primate genomes. It thus suggests a broader range of targets for retrotransposition than previously known, expanding our understanding of ME activity in primate genomes.

 In general, the manuscript has a smooth flow in its writing, presenting interesting and compelling results. Here are some suggestions for improvement:

  

1. While the term “retro-DNAs” is appropriate, considering them as "a new type of MEs" may be misleading. Similar to retro-copies or retro-genes, retro-DNAs are by-products of L1-mediated retrotransposition. In theory, any polyadenylated RNAs could be recognized by reverse transcriptase encoded by L1s  [Ref-1]. However, these sequences cannot autonomously transpose as MEs. I agree with the first reviewer that the authors should better tune down their statement.
2. Consistency in formatting TE names is crucial. Ensure all TE names are italicized throughout the manuscript, as some inconsistencies were noted (e.g., hAT and TcMar superfamilies, hAT-Tip100, and Tigger7 on page 10).
3. Figure 7 was not cited in the manuscript, and it was redundant with Figure 8A. Please delete Figure 7 and reorder the Figures.
4. Is hAT-Trip100 is a typo of *hAT-Tip100*? Please check the writing.
5. Most L1copies are 5’-truncated, while their 3’ ends are necessary for the recognition by reverse transcriptase [Ref-2]. This is another important feature of retroposition by L1s. I noticed that the authors identified retro-DNAs and their parent sites. Can they further check whether retro-DNAs are from the 3’ ends of their parent sites? This will provide additional evidence supporting L1-mediated retro-DNA formation.

Is the work clearly and accurately presented and does it cite the current literature?

Yes

If applicable, is the statistical analysis and its interpretation appropriate?

Yes

Are all the source data underlying the results available to ensure full reproducibility?

Yes

Is the study design appropriate and is the work technically sound?

Yes

Are the conclusions drawn adequately supported by the results?

Yes

Are sufficient details of methods and analysis provided to allow replication by others?

Yes

Reviewer Expertise:

Transposon, TE-mediated gene duplication, genome evolution, new gene evolution

I confirm that I have read this submission and believe that I have an appropriate level of expertise to confirm that it is of an acceptable scientific standard, however I have significant reservations, as outlined above.

## References

[1] : The power of. *J Genet Genomics* .2023;50(7) : 10.1016/j.jgg.2023.04.003 462-472 10.1016/j.jgg.2023.04.003 37068629

[2] : Analysis of 5' junctions of human LINE-1 and Alu retrotransposons suggests an alternative model for 5'-end attachment requiring microhomology-mediated end-joining. *Genome Res* .2005;15(6) : 10.1101/gr.3421505 780-9 10.1101/gr.3421505 15930490 PMC1142468

# Reviewer report: Reviewer response for version 1

**DOI:** 10.5256/f1000research.142770.r251957

**Published:** 2024-03-26

**Authors:** Rene Massimiliano Marsano

**Affiliations:** 1 Università degli Studi di Bari "Aldo Moro", Bari, Italy

In this manuscript, the Authors conduct a comparative analysis of 10 primate genomes unveiling the identification of a new type of mobile elements, which they call “retro-DNAs”, displaying combined features of two Class I (non-LTR retrotransposons) and Class II (DNA transposons) elements.

 The Authors initially annotated DNA transposons (remnants) in the analyzed genomes and subsequently have characterized a subset of sequences featured by an unusually long TSD and a poly-A tail. Furthermore, the Authors inferred the lineage specificity of retro-DNA and determine the expression level of retro-DNA sequences identified.

 The manuscript is well-written and easy to read in all its parts.

 Find below a list of major issues.

 - While retro transposition can be considered as a way to spread transposable elements fragments around the genome and the information provided in this manuscript is (to my knowledge) new, I would be cautious in defining retro-DNAs as "a new type of non-autonomous non-LTR retrotransposons". retro position of expressed sequences is a common process and indeed, we do not define processed pseudogenes this way. I would therefore recommend modifying such a conclusion to avoid overstatements and misinterpretation of the result.

 - The expression analysis shows that some retro-DNA sequences are expressed in the testis. This finding compatible with what is usually seen for retrotranscribed pseudogenes?  Testis activation of pseudogenes is well-documented, suggesting potential functional roles. Many pseudogenes are activated in the testis [1],[2]. In the case of TEs (and especially non-functional TEs) testis expression may evolve new regulation functions as observed in other transposition systems which are subjected to piRNA regulation.

 - I cannot see the supplementary files associated to the manuscript.

Is the work clearly and accurately presented and does it cite the current literature?

Yes

If applicable, is the statistical analysis and its interpretation appropriate?

Yes

Are all the source data underlying the results available to ensure full reproducibility?

Yes

Is the study design appropriate and is the work technically sound?

Yes

Are the conclusions drawn adequately supported by the results?

Partly

Are sufficient details of methods and analysis provided to allow replication by others?

Yes

Reviewer Expertise:

transposable elements, genetics

I confirm that I have read this submission and believe that I have an appropriate level of expertise to confirm that it is of an acceptable scientific standard, however I have significant reservations, as outlined above.

## References

[1] : Origins, evolution, and phenotypic impact of new genes. *Genome Res* .2010;20(10) : 10.1101/gr.101386.109 1313-26 10.1101/gr.101386.109 20651121 PMC2945180

[2] : Structural and Comparative Analyses of Insects Suggest the Presence of an Ultra-Conserved Regulatory Element of the Genes Encoding Vacuolar-Type ATPase Subunits and Assembly Factors. *Biology (Basel)* .2023;12(8) : 10.3390/biology12081127 10.3390/biology12081127 37627011 PMC10452791

# Response

**Published:** 2024-04-12

**Authors:** Ping Liang

**Affiliations:** Brock University, Canada

Dear Dr. Marsano,

 Thank you very much for taking the effort to review our manuscript and for your overall positive review and constructive comments for improving the manuscript. We would like to offer our responses below to address the concerns you have raised and made some adjustments in our new version, and we would very much welcome your re-reading and approval.

 First, regarding your caution in our claiming retro-DNAs as “a new type of retrotransposon”, we would agree with you that many other types of DNAs, notably the mRNA of genes as processed pseudogenes or retrogenes, are not named as new types of retrotransposons in addition to LINEs/L1, SINEs/Alus and SVAs in the human genome. Very likely, we would agree with each other that a new type of retrotranspons (non-LTR specifically in our case), in addition to have the expected sequence features (i.e., polyA taile), would also have the ability to continue to retrotranspose like other non-autonomous non-LTR retrotransposons, such as Alus and SVAs, at least as a group, but not necessarily every individual copies. In other words, some of the retrotransposed copies (beyond the original copies) are able to be transcribed and retrotranspose. This extra feature would distinguish it from processed pseudogenes. Following this reasoning, we demonstrated that some of the retro-DNA copies have the capacity to transcribe or be transcribed and served as the parent copies of additional copies (Table 5 and Fig. 9A). Certainly, we realize the limitation of our data by lack of experimental data to the presence of demonstrate capacity of these copies, and ideally this is to be demonstrated by using the established retrotransposition assay (refs). However, this is beyond our reach as a bioinformatics-oriented research group, and it is our hope that by reporting such potential of these retro-DNA elements, interest can be stimulated by other groups to follow up with experimental verification.  Considering limitation of our research data, we have adjusted the relevant point in our conclusion as “retro-DNA could be established as a new type of non-LTR retrotransposons if their intrinsic L1-based transposition capacity can be experimentally approved.”

 Second, regarding our use of testis tissues for expression analysis of these retro-DNAs, our rationale is related to above point, their potential as parent copies of transmissible retro-DNAs, since only they need to be expressed in gamete cells for this to happen. It is true that a lot of genes show sporadic expression beyond their regular expression pattern in other differentiated tissue, likely attributed to the unique epigenetic profile during spermatogenesis. But this may likely also be the attributing factor for the generally higher levels of germline retrotransposition of all known retrotransposons over somatic retrotransposition, with the former responsible for all transmitted retrotransposition events observed in the genomes. For this reason, even though we did include a mixed tissues sample and blood, with the latter as a widely available somatic tissue, in our expression analysis and showed a certain level of their expression, we thought it would be more meaningful to examine their expression in germline tissues. We would like to also include ovary as the female germline tissue, but it is unavailable across multiple species. For these reasons, we would like to argue that our focus on expression in testis tissue is justifiable.  If you still have some concerns, we would welcome some specific suggestions for improving our writing.

 Lastly, you mentioned that you could not see the supplementary files associated to the manuscript. However, I check the link to the file, https://identifiers.org/biostudies:S-BSST1030,  under the “Data availability” section, is working properly. On this linked page, there are 3 listed files you can click and download or you can choose to download all files (a button below the file list). We wonder if you could check it again and let us know if you can access properly. This is one of the public data repositories recommended by the journal.

 Thank you in advance for your second read and further comments or full approval.

 Sincerely,

 Ping Liang
